# *Rickettsia parkeri* Strain Atlantic  Rainforest in Archived *Amblyomma geayi* from Three-Toed Sloth (*Bradypus tridactylus*) in Manaus, Brazil

**DOI:** 10.3390/ani15182645

**Published:** 2025-09-09

**Authors:** Rafaela Moreira, Guilherme Moreira, Mahima Hemnani, Carlos Augusto Rodrigues do Nascimento, Sergio Luís Gianizella, João Rodrigo Mesquita, Patrícia Ferreira Barradas

**Affiliations:** 1 ICBAS—School of Medicine and Biomedical Sciences, Porto University, 4050-313 Porto, Portugal; rafaelasimaomoreira@gmail.com (R.M.); gmoreiravet@gmail.com (G.M.); hemnanimahi@gmail.com (M.H.); jmesquita@outlook.com (J.R.M.); 2 Laboratório de Biologia Celular e Helmintologia ‘Profa Dra Reinalda Marisa Lanfredi’, Instituto de Ciências Biológicas, Universidade Federal do Pará, Guamá, Belém 66075-110, PA, Brazil; augustocrbio@gmail.com; 3 Laboratory of Zoology, Department of Biology, Institute of Biological Sciences, Federal University of Amazonas, Manaus 69067-005, AM, Brazil; slgiani@ufam.edu.br; 4 Centro de Estudos de Ciência Animal (CECA), Instituto de Ciências, Tecnologias e Agroambiente (ICETA), Universidade do Porto (UP), Rua D. Manuel II, Apartado 55142, 4051-401 Porto, Portugal; 5 Associate Laboratory for Animal and Veterinary Science (AL4AnimalS), 1300-477 Lisboa, Portugal; 6 CECAV—Veterinary and Animal Research Center, University of Trás-os-Montes e Alto Douro, Quinta de Prados, 5000-801 Vila Real, Portugal; 7 TOXRUN—Toxicology Research Unit, University Institute of Health Sciences, CESPU, CRL, 4585-116 Gandra, Portugal

**Keywords:** *Rickettsia parkeri*, *Amblyomma*, three-toed sloth, wildlife, *omp*B gene, *glt*A gene, Manaus

## Abstract

In the Brazilian Amazon biome, there has been a rise in human spotted fever cases, but still significant knowledge gaps regarding the diversity and epidemiology of the tick–host–*Rickettsia* relationship. In the herein study, rickettsiae were investigated in ticks from captured live wild hosts in the Amazon biome by PCR targeting a partial sequence of *omp*B and *glt*A genes. All 343 ticks were morphologically identified as belonging to five species of the genus *Amblyomma*. *Amblyomma varium* (*n* = 24, 7%) were collected from a *Rhinella marina* and a *Bradypus tridactylus*. *Amblyomma geayi* (*n* = 51, 15%) were collected from two *Bradypus tridactylus*. *Amblyomma goeldii* (*n* = 116, 34%) were collected from three *Tamandua tetradactyla*. *Amblyomma dissimile* (*n* = 131, 38%) were collected from two *Boa constrictor*. *Amblyomma humerale* (*n* = 21, 6%) were collected from a *Chelonoidis* spp. Four *A. geayi* ticks (one female and three males) collected from a three-toed sloth (*B. tridactylus*) in 2015 were found to be positive for *Rickettsia parkeri* strain Atlantic Rainforest. The molecular findings herein have confirmed that *R. parkeri* spotted fever may occur in the Amazon Rainforest associated with *A. geayi*, expanding the geographical distribution of the *R. parkeri* strain to the Central Amazon Rainforest.

## Simple Summary

An exploratory study was conducted to detect *Rickettsia* bacteria in ticks previously collected between 1982 and 2015 from various wild animals in the Brazilian Amazon. A total of 343 ticks were obtained from wild vertebrate hosts including *Rhinella marina*, *Bradypus tridactylus*, *Tamandua tetradactyla*, *Boa constrictor*, and *Chelonoidis* spp. All ticks were morphologically identified as belonging to five *Amblyomma* species: *A. varium* (7%), *A. geayi* (15%), *A. goeldii* (34%), *A. dissimile* (38%), and *A. humerale* (6%). Molecular screening targeting partial sequences of the *omp*B and *glt*A genes was performed using PCR assays. *Rickettsia parkeri* strain Atlantic Rainforest DNA was detected in four *A. geayi* ticks (one female and three males) collected from a three-toed sloth (*Bradypus tridactylus*) in 2015. These findings confirm that *R. parkeri* infection occurs in the Central Amazon and is associated with *A. geayi*, thereby expanding the known geographical range of this pathogenic strain. The findings provide new data on the enzootic cycle of *R. parkeri* in the Amazon and reinforce the need for continued surveillance of tick-borne rickettsioses in the region.

## 1. Introduction

Arthropod-borne pathogens represent a significant threat to humans, livestock, companion animals, and wildlife. Geographical dispersion and an increase in the population of ticks, a known vector for many human and animal pathogens, have been observed. Factors, such as climate change, urbanization, deforestation, habitat destruction, and conservation measures favoring certain tick species contribute to this tendency [1].

Brazil’s tick fauna comprises 77 valid species, 53 Ixodidae and 24 Argasidae [2]. Most of them are primarily associated with mammalian hosts, but also with reptiles, amphibians, and birds [3]. Ticks of the *Amblyomma* genus are currently represented by 137 valid species, approximately 20% of the Ixodidae family [4]. In Brazil, the genus *Amblyomma* is the most numerous, with 33 species [5] comprising 44% of the Brazilian tick fauna. Among the many known tick-borne pathogens, intracellular bacteria of the genus *Rickettsia* are known for their potential for pathogenicity [6]. In recent years, infections caused by rickettsial agents have emerged in certain areas of Brazil [7]. *Rickettsia rickettsii*, *R. massiliae*, and *R. parkeri* complex tick-borne spotted fever rickettsioses have been described in South America [8,9,10]. *Rickettsia rickettsii* is the microorganism that causes Brazilian spotted fever, an acute febrile infection disease mainly concentrated in specific regions of Brazil, particularly in the south and southeast [11]. Brazilian spotted fever can manifest as a serious and lethal disease in humans [12], with *A. sculptum* and *A. aureolatum* being the main vectors.

*Rickettsia massiliae*, is a recognized human pathogen in Europe; however, in South America, it is presumed to be underdiagnosed or misdiagnosed, likely due to the extensive distribution of its vector, *Rhipicephalus* spp. *ticks* [9]. *Rickettsia parkeri* sensu lato (s.l.), is an emerging pathogen that causes spotted fever in some Atlantic Forest biome areas in the northeastern, southeastern, and southern Brazilian regions. *Amblyomma ovale* and *A. tigrinum* are the main vectors of this *Rickettsia* species [10,13].

The *R. parkeri* strain from the Atlantic Rainforest, a recently identified strain, has only been reported in the Brazilian Atlantic Forest biome. It is associated with acute, mild, and self-limiting symptoms in humans and is transmitted by the *A. ovale* complex (including *A. ovale* and *A. aureolatum*) [14,15].

In the Brazilian Amazon biome, there has been a rise in human spotted fever cases linked to tick bites, raising concerns about the ongoing interaction between ticks and humans [16]. Nonetheless, there are still significant knowledge gaps regarding the diversity and epidemiology of the tick–host–*Rickettsia* relationship. As such, the present study aimed to investigate the rickettsial agents associated with *Amblyomma* ticks collected from captured wildlife in the Amazon Rainforest, Brazil.

## 2. Materials and Methods

### 2.1. Tick Sample Collection

A total of 343 *Amblyomma* spp. ticks were collected between 1982 and 2015 in Manaus, situated in the heart of the Amazon Rainforest, at several sites: “Jardim Mauá”; Brazilian Institute of the Environment and Renewable Natural Resources; “Nova Cidade”; Amazonas Federal University campus; Manaus city; Manaus Industrial zone I and II, Amazon highway 010. Ticks archived at the Federal University of Amazonas (UFAM) were selected for this study. Arthropods were collected in wild hosts from the Amazon Rainforest, belonging to the following groups: amphibians, *Rhinella marina*, (*n* = 1); reptiles, *Boa constrictor* (*n* = 2) and *Chelonoidis* spp. (*n* = 1); and mammals, *Bradypus tridactylus* (*n* = 3) and *Tamandua tetradactyla* (*n* = 3). Most of the ticks collected originated from two Wildlife Screening Centers. The first is federal and located at the headquarters of the Brazilian Institute of the Environment and Renewable Natural Resources (IBAMA) in Manaus, Amazonas. The second center is municipal and operated within the Sauim Castanheira Wildlife Refuge (RVS), administered by the Environmental Secretariat of the municipality of Manaus.

The ticks were collected from live hosts following a visual inspection. The ticks were removed from the host using serrated tweezers and directly placed into vials containing 70% ethanol. The preserved ticks were then analyzed, sorted, identified, and cataloged in the Paulo Bührnheim Zoological Collection (CZPB), the scientific collection at the Institute of Biological Sciences, Federal University of Amazonas (UFAM), Manaus. All ticks were morphologically identified at the species level using morphological current keys for Brazilian ticks [17].

All 343 ticks were morphologically identified as belonging to the genus *Amblyomma* and comprised five species. Table 1 provides a summary of their distribution by species, host, and sex. All ticks were deposited in the CZPB, the scientific collection housed at UFAM, under the accession numbers CZPB-IX-00201, CZPB-IX-00591, CZPB-IX-000723, CZPB-IX-000672, CZPB-IX-00576, CZPB-IX-000097, CZPB-IX-00386, CZPB-IX-00635, CZPB-IX-00763, and CPZB-IX-00010 (Supplementary Materials).

### 2.2. DNA Extraction and Rickettsia Molecular Identification

To detect *Rickettsia*, the DNA of the 343 ticks was extracted and processed individually. Each tick was washed in a 10% bleach solution, rinsed in deionized water to remove residual bleach, dried on filter paper, and transferred to 1.5 mL tubes [18].

A modification of the QIAamp^®^ DNA Mini Kit (Qiagen, Valencia, CA, USA) was used to extract DNA from the ticks following previously described methods for nucleic extraction in ticks [19]. Briefly, on each tick a short incision was made in the ventral body with a sterile scalpel blade, and 420 μL of lysis buffer and 25 μL proteinase K solution were added to 1.5 mL Eppendorf tubes. The tubes were briefly mixed by vortexing for 30 s, centrifuged for 2 min at 6000 g, and then incubated at 57 °C for 15 min. A 350 μL aliquot of the recoverable supernatant was transferred to a fresh microcentrifuge tube and 350 μL of RTL buffer was added. The tubes were again briefly mixed by vortexing for 30 s, pulse centrifuged, and the next steps followed the QIAamp^®^ DNAMini Kit (Qiagen, Valencia, CA, USA) using an automated QIAcube (Qiagen GmbH, Hilden, Germany). Eluted DNA was immediately preserved at −20 °C until further analysis.

DNA was then tested for *Rickettsia* by two consecutive PCR protocols. Ticks were pooled (nine ticks per pool), assuring each pool consisted of ticks from the same species and host.

These pools were initially screened for spotted fever group *Rickettsia* using a conventional PCR targeting a 511 bp fragment of the outer membrane protein B (*omp*B) gene, as previously described (Table 2) [20]. DNA samples from positive pools were individually tested using the same assay.

Tick DNA positive for *Rickettsia* was further studied to confirm positive results and genetically characterize *Rickettsia* spp. For this, ticks were tested for a large fragment (806 bp) region of the citrate synthase (*glt*A) gene (Table 2) [21].

For all reactions, Xpert Fast Hotstart Mastermix (2X) with dye (Grisp, Porto, Portugal) was used and 5 μL of genomic DNA was added to a 25 μL final volume of the reaction mixture. The reactions were carried out in an automatic DNA thermal cycler 100 (Bio-Rad, Hercules, CA, USA), including negative (water) and positive controls. The PCR amplification products were visualized using Xpert green (Grisp, Porto, Portugal) fluorescence after electrophoresis in a 1.5% agarose gel at 100 V for 30 min.

### 2.3. Sequencing and Phylogenetic Analysis

All positive amplicons were purified with Exo/SAP Go—PCR purification kit (Grisp, Porto, Portugal), and sequencing was performed for both strands of PCR products at i3S (Institute for Health Research and Innovation—University of Porto). Sequence editing and multiple alignments were performed with the BioEdit Sequence Alignment Editor v7.1.9 software package, version 2.1 (Ibis Biosciences, Carlsbad, CA, USA), and further analysis was performed by comparison with the sequences available in the NCBI nucleotide database (http://blast.ncbi.nlm.nih.gov/Blast.cgi, accessed on 1 May 2024).

A phylogenetic tree was constructed for *Rickettsia* using nine reference strains and four strains identified in this study. Phylogenetic analysis was based on a 400 nt partial region of the *omp*B. The tree was constructed using MEGA X [22]. The optimal model in MEGA X software was chosen based on the lowest Bayesian Information Criterion (BIC) score, using the maximum likelihood based on the GTR + G model, and 1000 bootstraps were replicated. The phylogenetic tree was visualized and edited using the Interactive Tree of Life (iTOL) platform, providing a detailed graphical representation and annotation of the phylogenetic relations among the analyzed sequences.

## 3. Results

A total of 343 *Amblyomma* ticks were tested using a set of PCR assays targeting two rickettsial genes, specifically *omp*B and *glt*A. From the initial *omp*B screening, four *Amblyomma* ticks (1.17%; 95% confidence interval [CI]: 3.2–29.6) showed to be positive for the partial *omp*B region. These belonged to *Amblyomma geayi* ticks (one female and three males) collected from a three-toed sloth (*Bradypus tridactylus*) in 2015. Bi-directional sequencing showed all *omp*B sequences were 100% identical and nBLAST analysis supported the finding of *Rickettsia parkeri* strain Atlantic Rainforest. Further characterization of the *omp*B sequences showed the highest hit (99.59%) with *R. parkeri* strain Atlantic Rainforest (CP040325) isolated from an *A. ovale* tick in Colombia. The retrieved R. parkeri *ompB* sequences were assigned the following GenBank accession numbers: PP746843, PP746844, PP746845, and PP746846.

To further genetically characterize the *R. parkeri* strains, all *omp*B positive ticks were screened for the *glt*A region. The extracted DNA samples of the same four ticks produced amplicons of the expected size. Sequencing of the *glt*A region confirmed that all were 100% identical and confirmed the *omp*B classification as R. parkeri. Characterization of the *glt*A products showed the highest hit (100%) to *R. parkeri* strain Atlantic Forest (MK814824) retrieved from *A. ovale* in Veracruz, Mexico. The retrieved *glt*A sequences were assigned the following GenBank accession numbers: PP314177, PP347735, PP347736, and PP347737.

**Figure 1:**
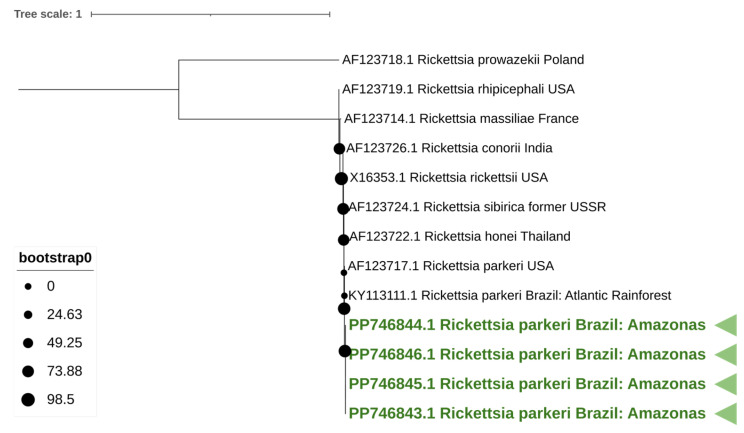
A phylogenetic tree of the *Rickettsia parkeri* strain Atlantic rainforest. A maximum likelihood method based on the GTR + G model phylogenetic tree was constructed based on *Rickettsia omp*B DNA sequences. Reliability of internal branches was assessed using the bootstrapping method (1000 replicates). *Rickettsia parkeri* strain Atlantic rainforest characterized in this study are indicated in green with the description of the GenBank accession number, sample number, *Rickettsia* species, and the country where it was found.

Phylogenetic analysis was subsequently conducted using the acquired sequences alongside nine reference strains. Samples from this study are indicated in green with the description of the GenBank accession number, sample number, host species, and the country where it was found. The analysis based on the *omp*B region confirmed its placement as *R. parkeri* (Figure 1).

## 4. Discussion

The recording of spotted fever cases in the Notifiable Diseases Information System (SINAN), alongside the implementation of rickettsiosis environmental surveillance in Brazil, has enabled the unambiguous identification of deaths from the disease, explaining the increase in human spotted fever cases associated with tick bites [7]. However, there is still a lack of knowledge regarding the epidemiology of tick-borne rickettsiosis in the Amazon biome. To contribute to this knowledge, the present study aimed to investigate the rickettsial agents associated with *Amblyomma* ticks collected from wildlife in the Brazilian Amazon Rainforest, Manaus. From the total 343 ticks of the *Amblyomma* genus studied, four (1.17%; 95% confidence interval [CI]: 3.2–29.6) showed to be positive for both the partial *omp*B and *glt*A regions, with both BLAST v2.17.0 (http://blast.ncbi.nlm.nih.gov/Blast.cgi, accessed on 1 May 2024) and phylogenetical analysis confirming the classification as *Rickettsia parkeri* strain Atlantic Rainforest.

*Rickettsia parkeri* sensu stricto, a member of the spotted fever group, was identified in 2004, as a cause of human infection in the United States [23]. A clinically identical infection was later reported in Brazil and attributed to the *R. parkeri* Atlantic Rainforest strain [15]. Since then, human clinical cases caused by the *R. parkeri* Atlantic Rainforest strain have emerged in many Brazilian regions [14,24], as well as in other countries such as Uruguay [25], Argentina [26], and Colombia [27].

In nature, *R. parkeri* strain Atlantic Rainforest has been reported in *A. aureolatum* [13], in *Dermacentor parumapertus*, and is most commonly associated with *A. ovale* [16]. This tick species, often collected from domestic dogs in Colombia, was previously identified as a host for the Atlantic Rainforest strain [28]. This strain is notable for causing mild rickettsiosis in Brazil, particularly in the southern state of Santa Catarina [24]. In this region, *A. ovale* and *A. aureolatum*, the most common ticks that bite humans, were found infected with *R. parkeri* strain Atlantic Rainforest in areas where human cases of rickettsiosis have been reported [29]. In the current study, all ticks testing positive for *R. parkeri* strain Atlantic Rainforest were identified as *A. geayi*, collected from a three-toed sloth (*B. tridactylus*) in Manaus. The three-toed sloth is included in the *Bradypodidae* family, genus *Bradypus*, which comprises four species: *Bradypus variegatus*, *Bradypus tridactylus*, *Bradypus torquatus*, and *Bradypus pygmaeus*. *Bradypus tridactylus* is commonly found in forest fragments of Manaus city, although it is a poorly studied species. They present unique characteristics, such as low and variable body temperature, sensitivity to environmental changes, and low resting metabolic rate, corresponding to 45% of the metabolic rate of an animal of the same size [30]. These animals are found in tropical forests, and studies have been conducted on their ticks and their potential risks [31].

In this study, ticks were also collected from *Rhinella marina*, *Boa constrictor*, and *Chelonoidis* spp. Amphibians and reptiles are abundant in Brazil, which ranks third globally in reptile fauna diversity. The Amazona state, covering the largest expanse of the Amazon Forest in Brazil, harbors 250 reptile species on its own [3]. Although previous papers have reported the presence of *Rickettsia* spp. in these animals [3,32,33], we were not able to detect *Rickettsia* spp. in ticks feeding on them.

The collared anteater, *Tamandua tetradactyla*, included in the *Myrmecophagidae* family, is extensively distributed across all Brazilian biomes [34]. Interestingly, they are frequently encountered in anthropogenic environments. Despite being classified as of least concern, their population is declining due to hunting and habitat loss [34]. Although there have been reports of *Rickettsia* spp. in these animals [35], in our study, we did not report *Rickettsia* in the ticks collected from this animal.

As far as we know, this report represents the first identification of *R. parkeri* strain Atlantic Rainforest in *A. geayi* ticks. These ticks are distributed in Central and South America [36] and in Brazil have been reported within the Amazon state [37]. The larval stage usually feeds on passerine birds while the nymph and mature stage commonly feed on sloths of the genera *Bradypus* and *Choloepus* (*Xenarthra*) [4,38].

This study has also screened other *Amblyomma* ticks for *Rickettsia* spp., namely in *A. goeldii*, *A. varium*, *A. dissimile*, and *A. humerale*. *Amblyomma goeldii* has a geographical distribution that appears restricted to the Amazonian region where males and females have been described [39]. There is no host record for *A. goeldii* immature stages; however, a nymph was described [39]. Nevertheless, several studies indicate that anteaters and large snakes are important hosts for their adult stage. In the state of Amazonas, *A. goeldii* has been associated with *Tamandua tetradactyla* [40], which is consistent with our findings.

*Amblyomma varium* is a neotropical tick popularly known as the sloth’s giant tick. It is currently found in several Brazilian states, namely Amazonas [31]. Immature stages (larva and nymph) were described in Rodentia: *Echimyidae*, Passeriformes (several families) and Piciformes: *Bucconidae* [38]. During the adult stage it is found almost exclusively in mammals of the *Bradypodidae* and *Megalonychidae* families of the superorder Xenarthra. Interestingly, in our study, it was found that *A. varium* adult ticks were parasitizing *Rhinella marina*. Although rickettsiae were not detected in *A. varium* in this study, this tick species has previously been reported to be infected with *Rickettsia bellii* [40].

*Amblyomma dissimile* primarily uses amphibians and reptiles as hosts for its larvae, nymphs, and adults [41], which follows our findings. This tick species has a broad geographic distribution, with reports of human, cattle, and sheep infestation [42]. None of the *A. dissimile* ticks examined in our study showed evidence of rickettsial infection in contrast to other studies, which reported the presence of *Rickettsia bellii* and *Rickettsia* sp. strain Colombianensi [43].

*Amblyomma humerale* is endemic to South America, with Brazilian reports of this tick species in several states of Amazonia [44]. Consistent with our findings, previous host records indicate that the adult stage parasitizes tortoises [44]. Nonetheless, it has been reported that a domestic dog, in the Rio Grande do Sul state, was infested with this tick species, as well as a toad *Bufo arenalis* in the state of São Paulo. While *Rickettsia* was not detected in the *A. humerale* examined in this study, previous reports have shown that this tick species can be infected with *R. bellii* and *Rickettsia amblyommatis* [45].

The Amazon’s appeal to tourists is often attributed to the opportunity to observe and interact with its diverse wildlife. This study confirms the presence of *R. parkeri* strain Atlantic Rainforest in Amazonian wildlife, and their proximity to humans may facilitate the transmission of this *Rickettsia* to people.

The findings of this study indicate that *R. parkeri* strain Atlantic Rainforest circulates in the Amazon Rainforest associated with *A. geayi*.

There is no reliable evidence to suggest that *A. geayi* causes human parasitism, nor is there any indication that this tick species could transmit *R. parkeri* spotted fever to humans. Nonetheless, it might serve as a vector for other hosts, and for this reason, further studies are imperative for a better understanding of the enzootic cycle of *R. parkeri* strain Atlantic Rainforest in the Amazon region.

## 5. Conclusions

This research expands our understanding of the geographical distribution of *Rickettsia parkeri* strain Atlantic Rainforest in the Amazon biome and on the potential vector spectrum. It also highlights the ecological complexity and public health relevance of tick-borne diseases in this region while emphasizing the need to develop effective strategies to prevent and control tick-borne diseases in the Amazon Rainforest and beyond.

## Figures and Tables

**Table 1:** Tick species collected, number of specimens, host species, and sex distribution. The total number of samples (N) and their corresponding percentages refer to the proportion of each tick species relative to the total number of collected specimens. The number of host individuals is indicated in parentheses in the “Host(s)” column.

Species	Samples (N)	%	Host(s)	Females	Males
*Amblyomma varium*	N = 24	*7*	*Rhinella marina* and *Bradypus tridactylus*	3	21
*Amblyomma geayi*	N = 51	15	*Bradypus tridactylus* (2)	5	46
*Amblyomma goeldii*	N = 116	34	*Tamandua tetradactyla* (3)	23	93
*Amblyomma dissimile*	N = 131	38	*Boa constrictor* (2)	57	74
*Amblyomma humerale*	N = 21	6	*Chelonoidis* spp.	2	19

**Table 2:** Primer sequences.

Target Gene	Primer Sequence (5′ to 3′)	Size (bp)	References
*omp*B	rompB OF: GTAACCGGAAGTAATCGTTTCGTAA rompB OR: GCTTTATAACCAGCTAAACCACC	511	Choi et al., 2005 [20]
*gltA*	CS 415: GCTATTATGCTTGCGGCTGT CS 1220: TGCATTTCTTTCCATTGTGC	806	de Sousa et al., 2005 [21]

## Data Availability

The original contributions presented in this study are included in the article. Further inquiries can be directed to the corresponding author.

## Supplementary Materials

The following supporting information can be downloaded at https://www.mdpi.com/article/10.3390/ani15182645/s1; List of ixodid ticks, hosts, locations, collection dates, and cataloging code at CZPB.
